# PatJAZ6 Acts as a Repressor Regulating JA-Induced Biosynthesis of Patchouli Alcohol in *Pogostemon Cablin*

**DOI:** 10.3390/ijms20236038

**Published:** 2019-11-30

**Authors:** Xiaobing Wang, Xiuzhen Chen, Liting Zhong, Xuanxuan Zhou, Yun Tang, Yanting Liu, Junren Li, Hai Zheng, Ruoting Zhan, Likai Chen

**Affiliations:** 1 Joint Laboratory of National Engineering Research Center for the Pharmaceutics of Traditional Chinese Medicines, Key Laboratory of Chinese Medicinal Resource from Lingnan, Ministry of Education, Research Center of Chinese Herbal Resource Science and Engineering, Guangzhou University of Chinese Medicine, Guangzhou University of Chinese Medicine, Guangzhou 510006, China; 13570332896@163.com (X.W.); xiuzhenchan@163.com (X.C.); 13726857667@163.com (L.Z.); 15521166528@163.com (X.Z.); TY1216569833@163.com (Y.T.); sonicflyt@163.com (Y.L.); leejunren@163.com (J.L.); zhanrt@gzucm.edu.cn (R.Z.); 2 School of Pharmaceutical Sciences, Guangdong Food and Drug Vocational College, Guangzhou 510520, China; zhenghai2007@163.com

**Keywords:** PatJAZ6, jasmonic acid (JA) signaling pathway, *Pogostemon cablin*, patchouli alcohol, biosynthesis

## Abstract

The JASMONATE ZIM DOMAIN (JAZ) proteins act as negative regulators in the jasmonic acid (JA) signaling pathways of plants, and these proteins have been reported to play key roles in plant secondary metabolism mediated by JA. In this study, we firstly isolated one JAZ from *P. cablin,* PatJAZ6, which was characterized and revealed based on multiple alignments and a phylogenic tree analysis. The result of subcellular localization indicated that the PatJAZ6 protein was located in the nucleus of plant protoplasts. The expression level of *PatJAZ6* was significantly induced by the methyl jasmonate (MeJA). Furthermore, by means of yeast two-hybrid screening, we identified two transcription factors that interact with the PatJAZ6, the PatMYC2b1 and PatMYC2b2. Virus-induced gene silencing (VIGS) of *PatJAZ6* caused a decrease in expression abundance, resulting in a significant increase in the accumulation of patchouli alcohol. Moreover, we overexpressed *PatJAZ6* in *P. cablin*, which down-regulated the patchoulol synthase expression, and then suppressed the biosynthesis of patchouli alcohol. The results demonstrate that PatJAZ6 probably acts as a repressor in the regulation of patchouli alcohol biosynthesis, contributed to a model proposed for the potential JA signaling pathway in *P. cablin*.

## 1. Introduction

*Pogostemon cablin* (*P. cablin*), the medicinal part of which is dry whole grass, is a kind of Labiatae plant that has long been considered an important Chinese herbal medicine in Lingnan [1]. The main medicine produced from *P. cablin* is patchouli alcohol, which has been reported to have antibacterial [2], anti-inflammatory [3] and vasodilatory [4] properties, among others. On the one hand, patchouli alcohol may be mainly used in the perfume and cosmetics industry, and on the other hand, it may be frequently used for medical treatment. Due to the large market demand [5], an increasing number of scholars have begun to study *P. cablin*, especially the molecular synthesis mechanism of patchouli alcohol. Many investigations have focused on pharmacological effects in *P. cablin*; however, current knowledge on patchouli alcohol biosynthetic pathways is limited. In cultivation and production, the quality of patchouli is often related to planting light conditions, ambient temperature and different abiotic and biotic stresses. Previous studies in our research group found that these factors affected the expression of the patchouli alcohol synthase gene and caused significant differences in patchouli alcohol accumulation [6]. Subsequently, various exogenous hormone treatment experiments showed that patchouli alcohol synthesis was specifically induced by Methyl jasmonate (MeJA). A large number of studies have shown that different environmental signals stimulate plant synthesis of jasmonic acid [7], which affects the synthesis and accumulation of important secondary metabolites and their chemical reactions through JA signaling. Therefore, we predict that the biosynthesis process of patchouli alcohol may be highly dependent on the JA signaling. The pattern of JA signaling regulating the synthesis of secondary metabolites in medicinal plants has progressed in *Catharanthus roseus*, the main component of which is vinblastine [8]. In addition, this signaling also exists in tobacco (nicotine) [9].

JA is one of the most important signaling molecules in plants, governing responses to abiotic and biotic stresses, as well as in secondary metabolite biosynthesis, signal transduction, stress response [10], growth and development [11]. The JASMONATE ZIM DOMAIN (JAZ) proteins are not only an important component of the JA signaling pathway but also a node that links different signaling pathways in plants [12]. The *N*-terminus of the JAZ contains a weakly conserved N-terminal (NT) domain. The ZIM domain contains a conserved TIF[F/Y]XG (TIFY) motif, and the Jas domain at the *C*-terminus is highly conserved and can interact with many proteins [13], such as various transcription factors (TFs). In addition, there are nuclear localization signals in the Jas domain, which causes JAZ proteins to have nuclear localization properties [14]. Thirteen JAZ proteins have been found in *Arabidopsis thaliana*, playing roles in growth, defense, and reproductive output [15], these proteins are regarded as repressors in the JA signaling pathway, and they interact with the MYC2 transcription factor and repress its function. MYC2 is the initial transcription factor of the JA response gene [16], but the JA induction of plants is controlled by CORONATINE INSENSITIVE 1 (COI1). JAZ family proteins have been identified as COI1 targets and repressors of MYC2 [17]. To date, JAZ proteins have been proven to repress the activity of TFs, and function as repressors in the JA pathway to mediate many developmental processes, including secondary metabolite synthesis.

As a pivotal regulator in the plant JA signaling pathway, JAZ proteins should play important regulatory roles in the biosynthesis of patchouli alcohol in *P. cablin*. However, little is known about the unambiguous roles of JAZ proteins in patchouli alcohol biosynthesis in *P. cablin*. In our previous work, we obtained 82,335 raw data of transcriptome using next-generation sequencing (NGS) technology from leaves of *P. cablin* treated with MeJA [18], 12 unigenes were recognized and identified as JAZ family genes, based on the expression levels of 12 genes under the induction of 300 µM MeJA and our previous screening experiment, we ultimately chose *PatJAZ6* (Unigene48011) as the research object, which may participate in the regulation of biosynthesis of patchouli alcohol.

In this study, the *PatJAZ6* gene from *P. cablin* was cloned and characterized, and the relative expression pattern analysis was performed. Then, the subcellular localization study of PatJAZ6 was performed. To further research on the interaction between PatJAZ6 and PatMYC2b1/PatMYC2b2, which was cloned and identified by our laboratory [18], we performed a Yeast two hybrid (Y2H) assay and Firefly Luciferase Complementation Imaging Assay (LCI) verification. Furthermore, to illuminate the roles of PatJAZ6 in patchouli alcohol biosynthesis, gene silencing induced by viruses and overexpression of *PatJAZ6* in plants was examined and analyzed. We ultimately elucidated a molecular conduction model between PatJAZ6 and PatMYC2b1/PatMYC2b2 in patchouli alcohol biosynthesis. The present study is the first to analyze the function of the *PatJAZ6* gene in *P. cablin*, which may be instructive for the study of JA signaling molecular mechanisms and secondary metabolites in *P. cablin*.

## 2. Results

### 2.1. Bioinformatics Analysis of PatJAZ6 from P. cablin

**Figure 1:**
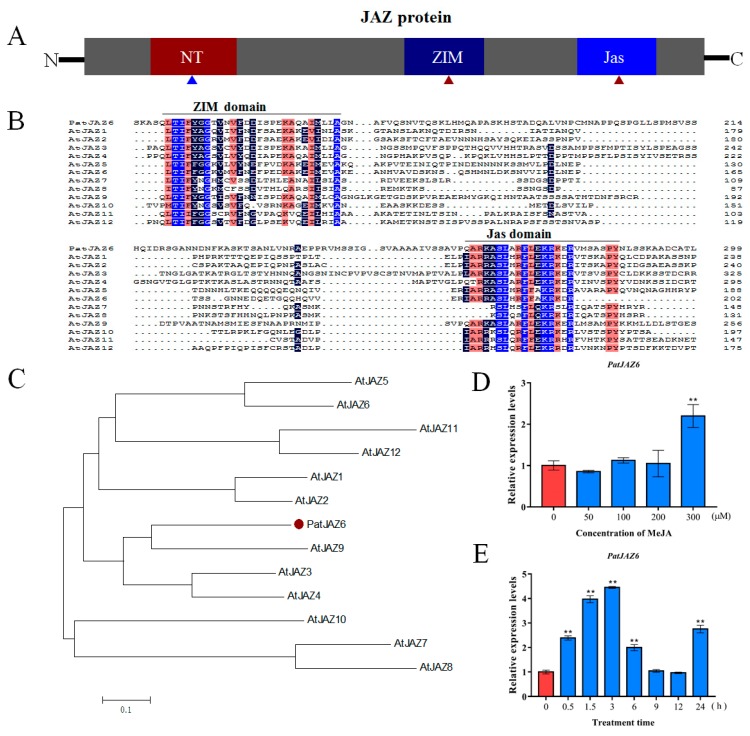
Bioinformatics analysis and expression profiles of *PatJAZ6*. (**A**) A schematic diagram of JAZ (Jasmonate ZIM domain, JAZ) protein. Red triangles represent conserved domains, blue triangle represents a weakly conserved NT domain. (**B**) Multiple alignments of PatJAZ6 with 12 JAZ proteins from *Arabidopsis thaliana*. AtJAZ1 (NP_564075.1), AtJAZ2 (NP_565096.1), AtJAZ3 (NP_974330.1), AtJAZ4 (NP_175283.2), AtJAZ5 (NP_001320905.1),AtJAZ6 (NP_001321693.1), AtJAZ7(NP_181007.1), AtJAZ8(NP_564349.1), AtJAZ9(AAL32593.1), AtJAZ10(NP_568287.1), AtJAZ11(NP_001190007.1), AtJAZ12 (NP_197590.1). (**C**) Phylogenic tree of PatJAZ6 with 12 JAZ proteins from *A. thaliana* was built using MEGA 7. The red solid dot represents PatJAZ6. (**D**) The relative expression of *PatJAZ6* was calculated after treatment with different concentrations of MeJA for 8 h, and the results were calculated according to the expression of *PatJAZ6* at 0 µM MeJA. (**E**) The relative expression of *PatJAZ6* was analyzed at different time points under 300 µM MeJA treatment. The results were calculated based on the expression of *PatJAZ6* at 0 h. (One-way ANOVA test; ** *p* < 0.01).

To determine the basic bioinformatics of *PatJAZ6*, several software and websites were used in this work. Analysis of the sequence revealed that *PatJAZ6* consists of 1375 bp with a 972-bp open reading frame (ORF). Amino acid alignment confirmed that PatJAZ6 is a member of the *JAZ* family. JAZ proteins contain three conserved domains, NT, ZIM, and Jas (Figure 1A).Multiple alignments of PatJAZ6 with 12 *JAZ* proteins from *Arabidopsis thaliana* showed that PatJAZ6 contained two conserved domains: the ZIM domain (TIFY motif), which is located near the *N*-terminus, and the Jas domain, which is near the *C*-terminal (Figure 1B). To further identify the characteristics of PatJAZ6, a phylogenetic tree was constructed using MEGA 7 software. The result illustrated that PatJAZ6 was highly homologous to AtJAZ9 (Figure 1C).

### 2.2. Expression Profiles of PatJAZ6 under MeJA Treatments

MeJA plays an important role in regulating secondary metabolite synthesis in a variety of plants. To detect whether *PatJAZ6* responds to MeJA, the relative expression of *PatJAZ6* was analyzed by qRT-PCR after treatment with different concentrations of exogenous MeJA for 8 h (Figure 1D). This analysis showed that 300 µM MeJA effectively induced the expression of *PatJAZ6* in *P. cablin*; therefore, 300 µM MeJA was selected as the lowest effective concentration to detect the expression of *PatJAZ6*. Different time points (0, 0.5, 1.5, 3, 6, 9, 12, and 24 h) were set after MeJA treatment to detect *PatJAZ6* expression. The results showed that MeJA effectively induced the expression of *PatJAZ6* in *P. cablin* (Figure 1E). Within 3 h of MeJA treatment, the expression levels of *PatJAZ6* increased rapidly and reached a maximum at 3 h, subsequently decreased at 6 h, and returned to the initial levels at 9 h and 12 h. After 24 h, the expression of *PatJAZ6* began to increase again, which is possible that expression of *PatJAZ6* is driven by circadian rhythms.

### 2.3. Subcellular Localization of PatJAZ6

**Figure 2:**
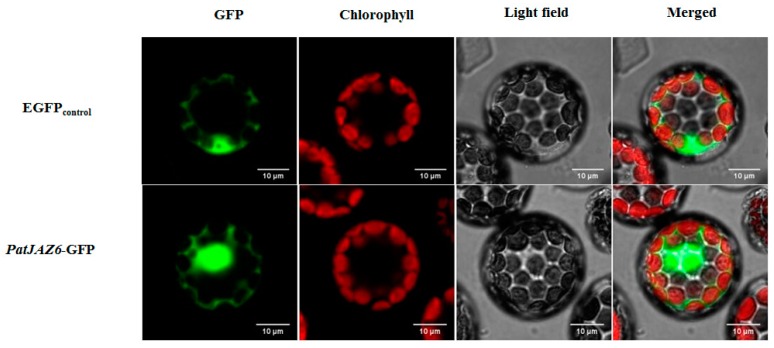
Subcellular localization of PatJAZ6 in *Arabidopsis* protoplasts. The open reading frame (ORF) without a termination codon was inserted into the vector named PAN580, Enhanced Green Fluorescent Protein (EGFP) was used as a control.

To determine the subcellular localization of PatJAZ6, the ORF of *PatJAZ6* without a termination codon was inserted into the *N*-terminus of the Green Fluorescent Protein (GFP) tag in vector PAN580. The recombinant plasmid was transformed into *A. thaliana* protoplasts by the polyethylene glycol (PEG-mediated method [19]. Subcellular localization results showed that PatJAZ6 was localized in the nucleus (Figure 2). We can infer that PatJAZ6 is highly likely to play functional roles in the nucleus, such as regulating transcription factors in the JA signaling pathway.

### 2.4. PatJAZ6 Protein Interacts with PatMYC2b1 and PatMYC2b2

**Figure 3:**
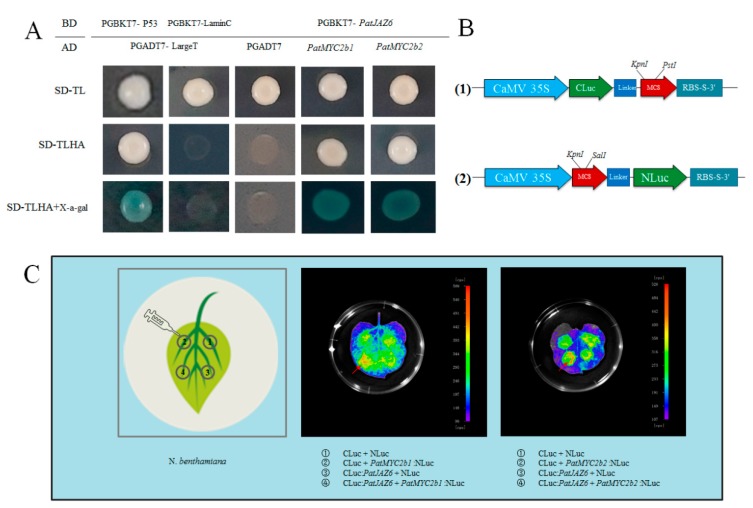
Protein interaction verification of PatJAZ6. (**A**) Yeast two-hybrid assay. Plasmids PGADT7-LargeT and pGBKT7-P53 were cotransformed into AH109 as the positive control. Plasmids PGADT7-LargeT and PGBKT7-LaminC were cotransformed into AH109 as the negative control. PGBKT7-*PatJAZ6* and pGADT7-TFs were cotransformed into *Saccharomyces cerevisiae* AH109 competent cells. The blue colonies represent the positive results. (**B**) (1) The complete ORF of *PatJAZ6* was inserted into the vector named PCAMBIA1300CLuc with the restriction enzyme sites *KpnI* and *PstI*. (2) The complete ORFs of *PatMYC2b1* and *PatMYC2b2* were inserted into the vector named PCAMBIA1300NLuc with the restriction enzyme sites *KpnI* and *SalI*, respectively. (**C**) Firefly Luciferase Complementation Imaging Assay. LCI images of *N. benthamiana* leaves coinfiltrated with the *Agrobacterial* GV3101-Psoup-p19 strains containing *PatMYC2b1*/*PatMYC2b2*:NLuc and CLuc:*PatJAZ6*. Arrow positions indicate where the signal is strongest.

Based on the reported research that JAZ proteins interact with some TFs [20], such as MYC2, which plays a regulated role in the JA signaling pathway, the ORFs of *PatJAZ6* and TFs (*PatMYC2b1*/*PatMYC2b2*) used in this study were cloned by the gene primers (Table S1) and fused to digested vectors PGBKT7 and pGADT7, respectively, in our research group. To explore whether PatJAZ6 can interact with PatMYC2b1 and PatMYC2b2, a Y2H screen was chosen to confirm the possible interaction relationship between them. Our screen results showed that pGBKT7-*PatJAZ6* + pGADT7-*PatMYC2b1* and pGBKT7-*PatJAZ6*+pGADT7-*PatMYC2b2* could grow normally on three types of screening plates and turned blue on SD/-Trp/-Leu/-His/-Ade/ plates containing X-α-Gal, which is consistent with the positive control, indicating that there is an interaction between PatJAZ6 and PatMYC2b1 /PatMYC2b2 in yeast systems, which implied that a relationship between PatJAZ6 and PatMYC2b1 /PatMYC2b2 may have existed in plants to regulate the biosynthesis of secondary metabolites (Figure 3A).

To verify whether this interaction exists in plants, further confirming the reliability of the Y2H results, a LCI was performed in *Nicotiana benthamiana* leaves by injecting *A. tumefaciens* GV3101-Psoup-p19 cultures containing recombinant constructs (Figure 3C). Injection positions ①, ② and ③, which represent different plasmid combinations, were set as negative controls. Injection positions ④ in LCI images showed large red areas, whereas ①, ② and ③ had almost no red areas, indicating that the signal at position ④ was significantly stronger than the control, revealing that PatJAZ6 and PatMYC2b1 /PatMYC2b2 have a strong interaction in *N. benthamiana,* implying a relationship between them most likely existed in *P. cablin*.

### 2.5. Effect on Patchouli Alcohol Biosynthesis by the Virus Induced PatJAZ6 Silencing

**Figure 4:**
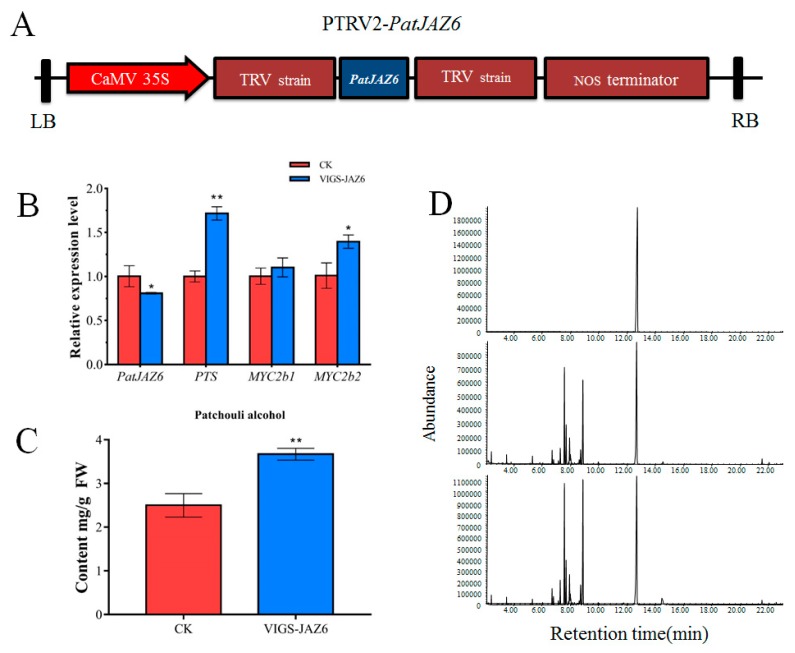
Analysis of virus-induced *PatJAZ6* silencing. (**A**) The *PatJAZ6* gene fragment (less than 500 bp) was cloned into the PTRV2 vector to form the PTRV2-*PatJAZ6*. (**B**) The corresponding mRNA expression level of VIGS-JAZ6 analyzed by real-time q-PCR. (**C**) The content of patchouli alcohol detected in control check (CK) and VIGS-JAZ6 leaves. (**D**) Gas Chromatography-Mass Spectrometer (GC-MS) chromatograms of samples from the standard of patchouli alcohol (top panel), CK (middle panel) and VIGS-JAZ6 (bottom panel) leaves showing abundance of patchouli alcohol. Asterisks indicate a significant difference from the control. (Student’s *t*-test; ** *p* < 0.01, * *p* < 0.05).

To investigate the roles of the PatJAZ6 protein in the JA signaling pathway affecting the synthesis of patchouli alcohol, Virus-induced gene silencing (VIGS) was selected to silence *PatJAZ6* to explore the effects of *PatJAZ6* on related genes and patchouli alcohol. The buffer containing a 1:1 ratio of PTRV1 and PTRV2 was set as a control. Leaf tissues were collected after *PatJAZ6* was silenced 14 days, which was used for qRT-PCR and Gas Chromatography-Mass Spectrometer (GC-MS) analysis. In the virus-induced *PatJAZ6* silencing, the relative expression level of *PatJAZ6* was clearly downregulated in comparison to the control (Figure 4B), while the expression of patchoulol synthase (*PTS*), which is the key enzyme for patchouli synthesis, was upregulated by approximately 80%. Moreover, the relative expression of *PatMYC2b1* and *PatMYC2b2* interacting with *PatJAZ6* were all increased, especially *PatMYC2b2*. The results of GC-MS showed that the content of patchouli alcohol in the VIGS-JAZ6 group (3.67 mg/g Fresh weight (FW)) was significantly higher than that in CK (2.5 mg/g FW), exhibiting an increase of 32% (Figure 4C). GC-MS chromatograms of samples from the standard of patchouli alcohol (top panel), CK (middle panel) and VIGS-JAZ6 (bottom panel) leaves showing abundance of patchouli alcohol (Figure 4D). The expression tendency of *PatJAZ6* was contrary to *PatMYC2b1* and *PatMYC2b2*, and based on the results of Y2H and LCI, we can speculate that PatJAZ6 plays a role as a transcriptional repressor in *P. cablin*. In addition, when *PatJAZ6* was silenced, patchouli alcohol synthesis was increased, which may be the result of a common increase in TFs and *PTS* gene expression.

### 2.6. Effect on Patchouli Alcohol Accumulation by the Overexpression of PatJAZ6

**Figure 5:**
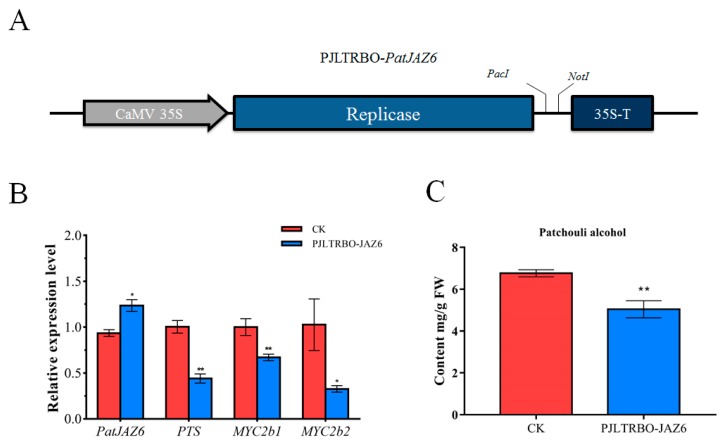
Overexpression analysis of *PatJAZ6*. (**A**) The *PatJAZ6* gene fragment was cloned into the PJLTRBO vector to form the PJLTRBO-*PatJAZ6* construct with the restriction enzyme sites *PacI* and *NotI*. (**B**) The corresponding mRNA expression level of PJLTRBO-*PatJAZ6* analyzed by real-time q-PCR. (**C**) The content of patchouli alcohol detected in CK and PJLTRBO-*PatJAZ6* leaves. Asterisks indicate a significant difference from the control (Student’s *t*-test; ** *p* < 0.01, * *p* < 0.05).

To confirm that PatJAZ6 plays a role as a transcriptional repressor in *P. cablin*, based on the VIGS-PatJAZ6 experiment, we hypothesized that overexpression of *PatJAZ6* may show opposite results to gene silencing of *PatJAZ6*. The empty PJLTRBO vector was transformed into GV3101-Psoup-p19 as CK. In the *PatJAZ6*-overexpressing *P. cablin* leaves, *PatJAZ6* expression was upregulated in comparison to the CK, while the transcripts of *PTS*, *PatMYC2b1* and *PatMYC2b2* were all reduced at different levels. Among these genes, *PatMYC2b2* showed the most significant decrease, exhibiting a nearly 70% reduction (Figure 5B). The content of patchouli alcohol in PJLTRBO-PatJAZ6 was determined by GC-MS, consistent with the gene expression profile of *PTS*, *PatMYC2b1* and *PatMYC2b2*. The accumulation of patchouli alcohol in *PatJAZ6* overexpressing appeared to decrease. Overexpression of *PatJAZ6* produced lower levels of patchouli alcohol (5.04 mg/g FW) compared with the control (6.76 mg/g FW) (Figure 5C). The results described above confirmed our conjecture, indicating that PatJAZ6 may repress the biosynthesis of patchouli alcohol.

## 3. Discussion

Patchouli alcohol, a natural tricyclic sesquiterpene compound that is a bioactive ingredient in *P. cablin* [21], is used worldwide in decorative cosmetics, toilet soaps, perfume industries [22] and medical treatments [23]. With the increasing market demand in the world, a metabolic engineering approach has been considered an effective approach to increase useful metabolites in medical plants, and several strategies have been reported to promote the industrialization process of patchouli alcohol production using *Saccharomyces cerevisiae* [24], and gene isolation and cloning in MVA and MEP pathways participating in patchouli alcohol biosynthesis have been reported [25]. In addition, full-length transcriptome data reported provide a valuable genetic resource in *P. cablin* [18]. Despite in-depth research on genes related to patchouli alcohol synthesis, little is known concerning the regulation of patchouli alcohol biosynthesis.

MeJA is an important plant endogenous hormone that is widely present in plants and regulates metabolic and developmental processes in plants [26]. Exogenous application of MeJA can stimulate the expression of defense genes and induce chemical defense in plants, including stimulating the synthesis of a series of secondary metabolites [27]. A growing number of reports indicate that the synthesis of many secondary metabolites in medicinal plants is increased under MeJA induction [28]. Our previous experiments showed that MeJA treatment on *P. cablin* leaves can significantly increase the accumulation of patchouli alcohol (Figure S1), but the specific molecular mechanisms involved have not been elucidated. We hypothesize that this effect may be related to JA signaling in plants and that the key factors of JA signaling are JAZ proteins. In our current research, the *PatJAZ6* gene was cloned from *P. cablin* and functionally identified as a repressor involved in patchouli alcohol biosynthesis. Bioinformatics analysis revealed that the *PatJAZ6* gene showed high homology with 12 JAZs from *A. thaliana Arabidopsis* and contained highly conserved ZIM and Jas domains, indicating that PatJAZ6 may have similar effects to previously reported JAZ proteins. The expression profiles under MeJA revealed that 300 µM MeJA was the lowest effective concentration to detect the expression of *PatJAZ6*, this concentration is higher in comparison with other JAZs, such as *NtJAZ* in tobacco [29] and *SmJAZ* in *Salvia miltiorrhiza* [30], that response to 100 µM MeJA. Subcellular localization results showed that PatJAZ6 was localized in the nucleus (Figure 2). This result is consistent with the characteristics of the Jas motif with nuclear localization. Previous studies on the subcellular localization of other JAZs also support this result [31].

JAZ proteins belong to ZIM-domain proteins, are located near the *C*-terminus, and have a highly conserved Jas motif of 26 amino acids. Studies have now determined that Jas motifs are involved in protein-protein interactions with MYC and COI1 [32]. In our present study, it was found that the PatJAZ6 protein can interact with PatMYC2b1 and PatMYC2b2 through a yeast two-hybrid (Y2H) screening method, which was consistent with previous reports that JAZ proteins interact with the MYC2 transcription factor [33]. Furthermore, an LCI assay was performed in *N. benthamiana* leaves, which further confirmed the interaction between PatJAZ6 and PatMYC2b1/PatMYC2b2 separately. The above data suggest that PatMYC2b1 and PatMYC2b2 transcription factors in *P. cablin* may be targets of the PatJAZ6 protein in *P. cablin* and play key regulatory roles in the accumulation of patchouli alcohol. Of course, there are other transcription factors involved in this synthesis process, which warrants further investigation.

VIGS is widely used to downregulate target genes in a majority of plants [34]. Tobacco rattle virus (TRV) is one of the most widely used viruses in VIGS technology [35]. The VIGS system constructed by this virus is rapidly applied to the model plant *N. benthamiana* [36] and such crops as pepper [37], cotton [38], and tomato [39]. For the silencing effect, some studies have shown that the silencing effect of the target gene fragment between 300 and 500 bp is the best [40]. Not long ago, the efficient VIGS system in *P. cablin* was established by our own laboratory (Figure S2A). According to other research reports, gene silencing in wild tobacco revealed that *NaJAZi* functions as a flower-specific jasmonate repressor that regulates JAs, TPIs, (E)-α-bergamotene and a defensin. Flowers silenced in *NaJAZi* are more resistant to tobacco budworm attack [41]. In addition, there are reports that knockdown of *AsJAZ1* expression through RNA interference led to decreased number of nodules, abnormal development of bacteroids, accumulation of poly-x-hydroxybutyrate (PHB) and loss of nitrogenase activity in legumes–rhizobia symbiosis [42]. However, in our experiments, gene silencing of *PatJAZ6* in *P. cablin* leaves did not exhibit a distinct phenotype, except for slight curling of the leaves, but an increase in the expression of *PTS*, *PatMYC2b1* and *PatMYC2b2* was observed, resulting in a significant increase in the accumulation of patchouli alcohol in the VIGS-JAZ6 group (3.67 mg/g FW) compared with the control in CK (2.5 mg/g FW).

Research on JAZs in cash crops, including *Oryza sativa* [43], *Glycine soja* [44] and *Gossypium hirsutum* [45], has progressed rapidly in the past several years. Overexpression of JAZs in these crops produces different phenotypes. For example, overexpression of *GsJAZ2* in soybean can increase the sensitivity of plants to salinity; overexpression of *GhJAZ2* in cotton impairs the sensitivity to JA, decreases the expression level of JA-response genes (*GhPDF1.2* and *GhVSP*) and enhances the susceptibility to *V. dahliae* and insect herbivory. However, we did not observe a significant phenotype in the *P. cablin* plants that overexpressed *PatJAZ6*, but we observed a decrease in the expression of *PTS*, *PatMYC2b1* and *PatMYC2b2*, resulting in a lower level of patchouli alcohol (5.04 mg/g FW) in PJLTRBO-JAZ6 compared with the control (6.76 mg/g FW). JAZ proteins have different functions, which may be due to the different roles of transcription factors interacting with JAZ proteins. Since it has been reported that JAZ protein may be involved in the development of glandular trichomes which, in turn, affects the synthesis of secondary metabolites in *Artemisia annua* [46]; therefore, whether the gene silencing or overexpression of *PatJAZ6* also affects the development of glandular trichomes in *P. cablin* requires further experimental confirmation.

**Figure 6:**
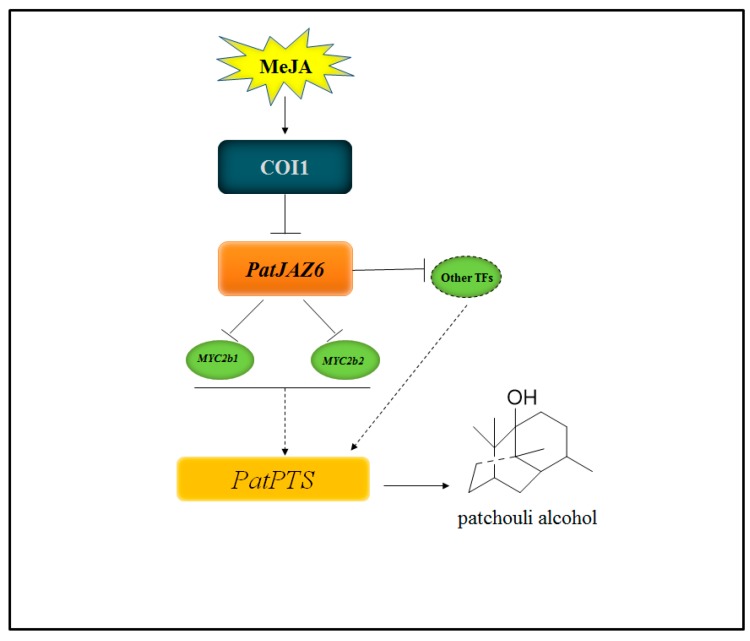
Model describing the function of *PatJAZ6* in JA-induced biosynthesis of patchouli alcohol. *PatJAZ6* acts as a repressor regulating JA-induced biosynthesis of patchouli alcohol in *Pogostemon cablin*.

In *S. miltiorrhiza* hairy roots, SmJAZ8 acts as a core repressor regulating JA-induced biosynthesis of salvianolic acids and tanshinones [30]. According to the results of our study, it is reasonable to speculate that PatJAZ6 may act as a repressor in patchouli alcohol biosynthesis. Based on the above experimental results, we first present a model for the JA signaling pathway in *P. cablin* (Figure 6).

The model clearly illustrates the connection of how *PatJAZ6* acts as a transcription factor suppressor regulating JA-induced biosynthesis of patchouli alcohol in *P. cablin*. However, further research is needed to determine whether PatJAZ6 interacts with other transcription factors and whether other JAZ proteins are involved in this signaling pathway. We also need to further explore the COI-JAZ-TFs model and how these proteins interact to regulate the synthesis of patchouli alcohol in *P. cablin.* Taken together, the results of this study help to elucidate the molecular regulation of JA signal-induced patchouli alcohol biosynthesis. Our work indicated that PatJAZ6 acts as a repressor in the regulation of patchouli alcohol biosynthesis. The discovery of the PatJAZ6 function points out a direction for the JA signaling pathway molecular mechanism and patchouli alcohol production in *P. cablin*.

## 4. Materials and Methods

### 4.1. Experimental Materials and Total RNA Extraction

The *P. cablin* plants were gathered from Yangjiang city, Guangdong Province, China. The cutting propagation method was used to obtain more seedlings that were used for the analysis of the expression patterns of *PatJAZ6* and content of patchouli alcohol in leaves. The seeds of *N. benthamiana* were kept in our laboratory and grown in flower pots in a growth chamber. Well-growing plant materials, which were cultured under a constant environment at 25 °C with a 16/8 h photoperiod treatment, were selected for our experiments. The vector plasmids, *E. coli* competent cells DH5α and *A. tumefaciens* competent cells used in this study were all kept in our own laboratory. Total RNA was extracted from *P. cablin* leaves with the GeneMark Plant Total RNA Purification Kit (GeneMarkBio, Taichung, Taiwan), and then a spectrophotometer (IMPLEN GNBH, Germany) was used to determine the RNA concentration and purity. cDNA synthesis was performed via oligo dT and stored at −20 °C for subsequent experiments.

### 4.2. MeJA Treatments

MeJA was purchased from Sigma-Aldrich, St. Louis, Missouri, The United States of America, USA, dissolved in ethanol, formulated into 50 mM mother liquor for later use. To screen for the best response concentration, *P. cablin* plants were sprayed with MeJA solution at 0, 50, 100, 200 and 300 µM concentrations containing 0.1% Tween-80, and leaf samples were collected at 8 h after MeJA treatments.

To investigate the effect of MeJA on *PatJAZ6* expression, *P. cablin* plants were sprayed in the morning with MeJA solution at a 300 µM concentration. Leaf samples were collected at time intervals of 0, 0.5, 1.5, 3, 6, 9, 12, and 24 h after MeJA treatment. All leaf samples were frozen with liquid nitrogen and stored in a −80 °C refrigerator for subsequent RNA extraction.

### 4.3. Bioinformatics Analysis of PatJAZ6

Bioinformatics analysis of *PatJAZ6* was performed using several bioinformatics software and websites. The ORF of *PatJAZ6* was determined using ORF finder (http://www.bioinformatics.org/sms2/orf_find.html), and 12 JAZ proteins from *A. thaliana* were searched from NCBI (https://www.ncbi.nlm.nih.gov/). MEGA v.7 software was used to construct the phylogenetic tree, and DNAMAN software was used to perform multiple sequence alignment.

### 4.4. Expression Patterns of PatJAZ6 by qRT-PCR

The expression patterns of *PatJAZ6* and related genes under different treatments were quantified by qRT-PCR. Plant tissues were gathered after various treatments, and total RNA was isolated. cDNA synthesis was performed using HiScript II QRT SuperMix for qPCR (Vazyme R222-01, Nanjing, China), and the CFX96TM Real-Time System was selected to carry out qRT-PCR analysis with ChamQ Universal SYBR qPCR Master Mix (Vazyme, Q711-02/03). qRT-PCR conditions were as follows: 95 °C for 3 min for one cycle, followed by 40 cycles of 95 °C for 10 s and 60 °C for 30 s. The relative expression levels of *PatJAZ6* and related genes were calculated based on the 2^−∆∆Ct^ method.

### 4.5. Subcellular Localization of PatJAZ6

The ORF of *PatJAZ6* without a termination codon was fused to the *N*-terminus of the vector pAN580-GFP tag with the restriction enzyme sites *SpeI* and *BamHI*. The recombinant plasmids PAN580-*PatJAZ6* were transformed into *Arabidopsis* protoplasts. The empty vector pAN580 was used as a negative control. The result of subcellular localization of PatJAZ6 was observed with ZEISS LSM 800 with Airyscan (ZEISS, Jena city, Germany).

### 4.6. Yeast Two-Hybrid Assays

The yeast two-hybrid (Y2H) screen was chosen to confirm possible TFs interacting with PatJAZ6. The ORF of *PatJAZ6* was cloned into the pGBKT7 vector to generate pGBKT7-*PatJAZ6*. Both the ORFs of *PatMYC2b1* and *PatMYC2b2* were inserted into the PGADT7 vector to form pGADT7-*PatMYC2b1* and pGADT7-*PatMYC2b2,* respectively. Plasmids PGADT7-LargeT and pGBKT7-P53 were cotransformed into *S. cerevisiae* AH109 competent cells as the positive control, while PGADT7-LargeT and PGBKT7-LaminC were cotransformed into AH109 as the negative control. Recombinant plasmids (pGBKT7-*PatJAZ6*+pGADT7-*PatMYC2b1*, pGBKT7-*PatJAZ6+* pGADT7-*PatMYC2b2* were cotransformed separately into AH109 cells and cultured on SD/-Trp/-Leu medium. Then, transformants were vaccinated on SD/-Trp/-Leu/-His/-Ade and SD/-Trp/-Leu/-His/-Ade/X-α-Gal to observe the interaction situation of *PatJAZ6* with *PatMYC2b1* and *PatMYC2b2*. All medium plates were incubated in an incubator at 29 °C for 3 days.

### 4.7. Firefly Luciferase Complementation Imaging Assay

To further understand the interaction between PatJAZ6 and PatMYC2b1/PatMYC2b2 in living plants, Firefly Luciferase Complementation Imaging Assay (LCI) was performed in *N. benthamiana* leaves. The complete ORF of *PatJAZ6* was inserted into the vector PCAMBIA1300CLuc with the restriction enzyme sites *KpnI* and *PstI*, while ORFs of *PatMYC2b1* and *PatMYC2b2* were inserted into the vector PCAMBIA1300NLuc with the restriction enzyme sites *KpnI* and *SalI,* respectively (Figure 3B). The *A. tumefaciens* strain GV3101-Psoup-p19 was transformed with recombinant constructs by the freeze–thaw method. The cultured bacteria solution was mixed at a ratio of 1:1, centrifuged and resuspended in buffer, adjusted optical density (OD) to 0.8–1.0, then placed at room temperature for 2–4 h, injected the back of *N. benthamiana* leaves with a needle-free syringe (Figure 3C), incubated for 12 h at 23 °C in the dark, and moisturized for 2–4 days at room temperature. The leaves were placed in MS solid medium with the leaves facing up, sprayed with 100 mM d-luciferin potassium salt, and kept in the dark for 6 min. A Berthold Technologies (LB983 NC100,Germany) was used to capture the images with an exposure time of 2 min.

### 4.8. Virus-Induced PatJAZ6 Silencing

The pTRV1 and pTRV2 vectors were kept in our laboratory and used in this study. A 418-bp fragment (Figure S2B) from the *PatJAZ6* ORF was cloned into the *EcoRI* and *BamHI* sites of the pTRV2 vector (Figure 4A). The resulting pTRV2-*PatJAZ6* constructs, PTRV1 and PTRV2, were transformed into *A. tumefaciens* GV3101. The mixture of *A. tumefaciens* cultures containing a 1:1 ratio of PTRV1 and PTRV2 or pTRV2-*PatJAZ6* was harvested by centrifugation and resuspended in infiltration buffer to obtain an OD of 1.0 and then incubated at room temperature for 2–4 h. Six-leaf-staged *P. cablin* plants were selected to infect with 1 mL needleless syringe on the abaxial side of leaves, and two to three leaves per plant needed infiltration. When *PatJAZ6* was silenced for 14 days, leaf tissues were collected and frozen for later use.

### 4.9. Overexpression Analysis

The complete ORF fragment of *PatJAZ6* was cloned into the PJLTRBO vector to form the PJLTRBO-*PatJAZ6* construct with the restriction enzyme sites *PacI* and *NotI* (Figure 5A). The recombinant plasmids PJLTRBO-*PatJAZ6* were transformed into GV3101-Psoup-p19, and the empty PJLTRBO vector was transformed into the same strain as the control. *A. tumefaciens* cultures were harvested and resuspended in infiltration buffer. For *P. cablin* leaf infiltration, the same injection method described in 4.8 was adopted. Samples were collected 4 days after plants were injected and frozen for later use.

### 4.10. Patchouli Alcohol Extraction and GC-MS Analysis

200 mg leaf tissues were ground frizzed in liquid nitrogen, 1.5 mL hexane was added into centrifuge tubes, ultrasonic for 30 min with 60 Hz and then heated under a 56 °C water bath for 1 h. After centrifugation, the supernatant was taken and passed through a 0.22-µm organic membrane as the test solution for GC-MS analysis using Agilent 7890B Gas Chromatograph with 5977A inert Mass Selective Detector (Agilent, California, USA). The gas chromatograph was equipped with an HP-5MS capillary column (30 m × 250 mm × 0.25 mm). The instrument was set to an initial temperature of 50 °C and maintained for 0 min. Then, the oven temperature was increased to 130 °C at a rate of 20 °C/min and then increased to 150 °C at a rate of 2 °C/min. The temperature was maintained at 150 °C for 5 min and later increased to 230 °C at a rate of 20 °C/min. The injection volume was 1 µL, and the injection port temperature was 230 °C. In addition, patchouli alcohol standards were purchased from NanTong FeiYu, China. All reagents used were analytical grade.

### 4.11. Agrobacterium Culture and Buffer Formulation

*A. tumefaciens* cultures were grown in the shaker with shaking (200 rpm/min) at 28 °C for 20–24 h. The infiltration buffer formulation as follows: 1 M 2-(4-morpholino)-ethane sulfonic acid; 1 M MgCl_2_ and 200 mM acetosyringone, dissolved in dimethyl sulfoxide.

### 4.12. Statistical Analysis

Statistical significance was determined by student’s *t*-test and one-way ANOVA.

## Supplementary Materials

Supplementary materials can be found at https://www.mdpi.com/1422-0067/20/23/6038/s1. Figure S1. MeJA treatment on *P. cablin* leaves can significantly increase the accumulation of patchouli alcohol. Figure S2. (A) Virus induced *PatPDS* silencing in *P. cablin*. (B) Electropherogram of cloning a 418 bp fragment of *PatJAZ6* into pTRV2 vector. Table S1: List of primers used in this study.
