# Supplementary material for: PatJAZ6 Acts as a Repressor Regulating JA-Induced Biosynthesis of Patchouli Alcohol in Pogostemon Cablin
Source: Int J Mol Sci. 2019 Nov 30;20(23):6038. doi: 10.3390/ijms20236038 (PMC6928788; doi:10.3390/ijms20236038)
Supplement: Supplementary file 1 [file ijms-20-06038-s001.pdf]

Article

PatJAZ6 acts as a repressor regulating JA-induced biosynthesis of patchouli alcohol in *Pogostemon cablin*

Xiaobing Wang[1], Xiuzhen Chen[1], Liting Zhong[1], Xuanxuan Zhou[1], Yun Tang[1], Yanting Liu[1], Junren Li[1], Hai Zheng[2], Ruoting Zhan[1], Likai Chen[1*]

Supplementary Materials:

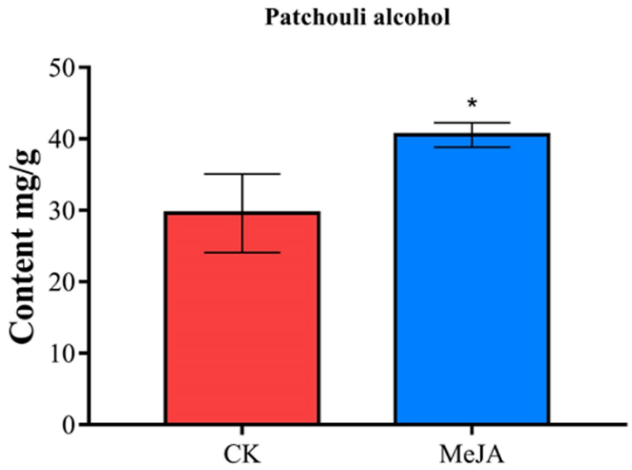

Figure S1. MeJA treatment on *P. cablin* leaves can significantly increase the accumulation of patchouli alcohol.

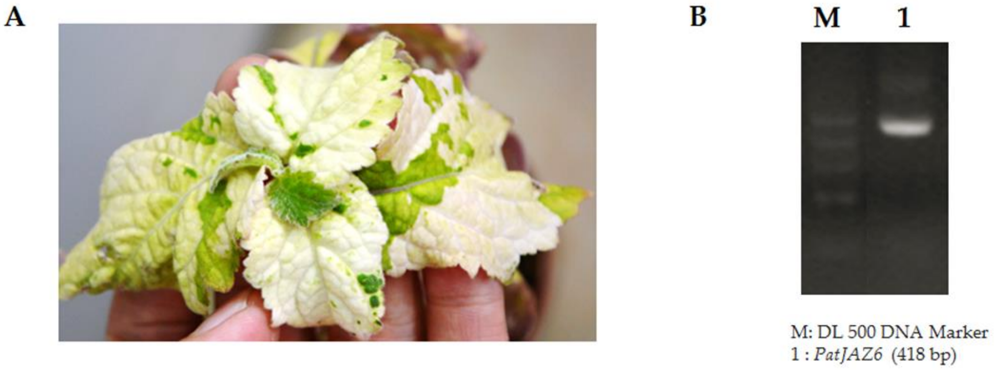

Figure S2. (A) Virus induced *PatPDS* silencing in *P. cablin.* (B) Electropherogram of cloning a 418 bp fragment of *PatJAZ6* into pTRV2 vector.

Table S1. List of primers used in this study.

Name	Sequence 5′ - 3′
PatJAZ6-F	TATGGAGAGAGATTTCATGGGGT
PatJAZ6-R	TTAATCTGTCTCCTTGGTGACAG
PatJAZ6-qPCR-F	GATCGGTCCGGTGCTAACAA
PatJAZ6-qPCR-R	TCATCACCCTCTCCTTTCGC
PAN580- *PatJAZ6*- F	aggacagcccagatcactagtATGGAGAGAGATTTCATGGGGTT
PAN580- *PatJAZ6*- R	gcccttgctcaccatggatccATCTGTCTCCTTGGTGACAGAGAGG
pGBKT7- *PatJAZ6*- F	atggccatggaggccgaattcATGGAGAGAGATTTCATGGGGTT
pGBKT7- *PatJAZ6*- R	ccgctgcaggtcgacggatccTTAATCTGTCTCCTTGGTGACAGAGA
1300Cluc-*PatJAZ6*- F	tacgcgtcccggggcggtaccATGGAGAGAGATTTCATGGGGTT
1300Cluc-*PatJAZ6*- R	atacgaacgaaagctctgcagTTAATCTGTCTCCTTGGTGACAGAGA
1300nLUC-*PatMYC2b1*-F	acgggggacgagctcggtaccATGATCGGTTACCGGACTCCC
1300nLUC-*PatMYC2b1*-R	cgcgtacgagatctggtcgacCTATCTACTCTCCCCCACCTTGG
1300nLUC-*PatMYC2b2*-F	acgggggacgagctcggtaccATGATCGGTTACCGGACTCCC
1300nLUC-*PatMYC2b2*-R	cgcgtacgagatctggtcgacCTATCTACTCTCCCCCACCTTGG
PTRV2-*PatJAZ6*-F	gtgagtaaggttaccgaattcGCAAATCATATCCAAGATGCAAAG
PTRV2-*PatJAZ6*-R	cgtgagctcggtaccggatccGTAGACCAGGGCTCTGTGGTGG
PJLTRBO-*PatJAZ6*-F	ttcgtgttcttgtcattaattaaATGGAGAGAGATTTCATGGGGTT
PJLTRBO-*PatJAZ6*-R	tcaagttgcaggaccgcggccgcTTAATCTGTCTCCTTGGTGACAGAGA
PatPTS-qPCR-F	CCAACCGCGAAACAAGTCC
PatPTS-qPCR-R	ATGCGTATTGCTCGTAGAT
